# Identification of novel targets of diabetic nephropathy and PEDF peptide treatment using RNA-seq

**DOI:** 10.1186/s12864-016-3199-8

**Published:** 2016-11-17

**Authors:** Ana Rubin, Anna C. Salzberg, Yuka Imamura, Anzor Grivitishvilli, Joyce Tombran-Tink

**Affiliations:** 1 Department of Neural and Behavioral Sciences, Penn State College of Medicine, Hershey, USA; 2 Functional Genome Sciences, Penn State College of Medicine, Hershey, USA; 3 Department of Ophthalmology, Penn State College of Medicine, Hershey, PA 17033 USA

**Keywords:** Diabetic nephropathy, PEDF P78 peptide, Global transcriptome changes, RNAseq, Canonical pathways, miRNA, Nhej1, Ept1, Mamdc4, Kdm4b

## Abstract

**Background:**

Diabetic nephropathy (DN) is a major complication of type1 and type 2 diabetes. Understanding how diabetes regulate transcriptome dynamics in DN is important for understanding the biology of the disease and for guiding development of new treatments.

**Results:**

We analyzed the kidney transcriptome of a DN mouse model, D2.B6-*Ins2* ^*Akita*^/MatbJ, before/after treatment with P78-PEDF. Age, weight, and gender-matched mice and wild-type (wt) littermates were treated at 6 weeks (early treatment) or 12 weeks (late treatment) of age for the duration of 6 weeks. Animals were implanted with an osmotic mini pump delivering 0.3 ug/g/day P78-PEDF or vehicle. Using RNA-seq, we identified14,316 transcripts (12,328 coding;1,988 non-coding) that were significant and reliably expressed (FPKM > =1) in diabetic kidneys. Expression of 1,129 (7.9%) including 901 coding genes was altered by diabetes with log2 fold changes (FC) between -86.2 and +86.0 (q < 0.05) compared to wt. Of these, 164 (14.5%) showed increased and 965 (85.5%) decreased expression with FC > 1.5. Coding genes with highest FC in diabetic kidneys include Nhej1 (32.04), Ept1 (8.6), Srd5a2 (-6.55), Aif1 (-6.05), and Angptl7 (-4.71).

Early and late stage diabetic groups receiving continuous infusion of P78 showed altered expression of 316/14,316 (2.2%) transcripts, including 121 coding genes compared to non-treated diabetic controls. Of these, 183 were upregulated and 133 downregulated with FC +50.9–-93.3 (q < 0.05). P78 reversed diabetes-induced changes in 138/1129 (12.2%) transcripts, including 49/901 (5.44%) coding genes. Nhej1 (-37.94), Tceanc2 (5.76), Ept1 (-4.45), Ugt1a2 (3.03), and Tmsb15l (-3.0) showed the highest FC with treatment. The DNA repair gene, Nhej1 with the greatest FC in diabetic kidneys was completely restored to control levels by both early and late P78 treatments. Expression of other coding genes regulated by diabetes with FC > =(+/-) 1.5 and completely reversed by P78 include Mamdc4, Kdm4b, Tmem252, Selm, and Hpd. RT and QRT-PCR validated expression of gene with FC > (+/-)2.0. Transcriptome changes were also observed between early and late-stage treatments.

Precursor non-coding miRNAs showed the highest fold changes in expression in the diabetic and P78 treatment groups. Several diabetic-induced changes were reversed in direction of expression by treatment including Gm24083, GM25953, miR1905, Gm25535, Gm27903, and miR196a1 with FC > =(+/-)20.

From Ingenuity pathway analysis (IPA), mitochondrial dysfunction, Nrf-2- mediated oxidative stress and renal injury pathways emerged as key mechanisms in DN. DN-enriching genes in these pathways were reduced in number or regulated in the opposite direction by treatment.

**Conclusions:**

Unique biomarkers and canonical pathways identified in this study may hold the key to understanding mechanisms of DN pathobiology with value for clinical translation. Our data suggest that mitochondrial dysfunction, genotoxicity and oxidative stress are principal events in DN and that P78-PEDF holds promise for its management.

## Background

The prevalence of diabetes in the world has been estimated at 2.8% in 2000, and is projected to increase to 4.4% by 2030 [1]. The disease currently affects approximately 8.3% of the US population, and is a leading cause of morbidity and mortality. Diabetes is a major cause of stroke, blindness, heart disease, and end-stage renal disease (ESRD) [2]. Among these sequelae, diabetic nephropathy (DN) is one of the most common complications of both Type 1 and Type 2 diabetes [3].

The pathophysiology of DN is complex and multiple mechanisms contribute to its development and outcome. Early hemodynamic changes and defective autoregulation of glomerular filtration rate lead to glomerular hyperfiltration and hyperperfusion [4]. Mechanisms involving glycosylation of tissue proteins [5], activation of Protein kinase C [6], and the Aldose reductase pathway [7] are believed to promote tissue damage, glomerular basement membrane thickening, glomerular hypertrophy and mesangial expansion. Other factors linked to the development and progression of DN include the expression of nephrin [8], inflammatory cytokines, vascular endothelial growth factor (VEGF) [9], lipid mediators [10], and reactive oxygen species [11]. Although, there is evidence that genetic predisposition influences the incidence and severity of DN, the low likelihood of identifying a single gene for the pathogenesis of DN has shifted research towards a multigene approach to understand mechanisms and etiology of the disease [4].

Current management of DN centers on preventing the development of risk factors such as hypertension, hyperglycemia, and dyslipidemia, early diagnosis, and antihypertensive therapy to reduce rate of decline in renal function [12]. Despite advancements in therapy, DN continues to be the most common cause of ESRD and requires dialysis in the U.S. [3]. The human and economic costs associated with ESRD raise the importance of risk factor reduction and the need to identify novel therapeutic targets to manage diabetes-induced kidney damage.

A large number of studies have now documented the protective role of pigment epithelium-derived factor (PEDF) against a wide range of oxidative and excitotoxic insults [13–15]. The neuroprotective, anti-angiogenic and anti-inflammatory properties of PEDF have been exploited in many preclinical therapeutic strategies [13] especially since the gene is expressed in several tissues [16] including the kidney where the highest expression is in the glomeruli [17]. In diabetes, serum levels of PEDF increase whereas tissue levels in the eye and kidney decrease in diabetic retinopathy and diabetic nephropathy respectively [18–20]. Such studies imply that restoring PEDF levels could reduce damage to tissues in diabetes. For example, increasing PEDF levels in the kidney by injecting an adenovirus expressing the protein significantly alleviates microalbuminuria in the early stages of diabetes [21].

Because PEDF is a large 50kD protein it has limited usefulness as a therapeutic agent in many cases. However, an active 44 amino acid fragment of PEDF (P78-PEDF) can block ischemic damage to retinal ganglion cells and reduce neuronal death, vascular abnormalities and inflammatory changes in a mouse model of diabetic retinopathy [22, 23]. We have shown that continuous infusion of this peptide in diabetic mice protects against development of diabetic nephropathy as indicated by reduced albuminuria, blood urea nitrogen, macrophage recruitment and expression of inflammatory cytokines and fibrotic markers, balanced nephrin expression, and decreased histological changes in diabetic kidneys [24]. In a proof of concept extension of those studies we confirmed that continuous systemic infusion of P78 blocks the progression of well-established diabetic nephropathy in the *Ins2* ^*Akita*^ mouse model of DN [25]. Here we used kidney samples obtained from the recently published proof of concept experiments [25] to study transcriptome changes in these non-treated and P78-treated diabetic nephropathy mice to identify novel gene candidates and pathways as regulators in the pathogenesis of DN.

## Methods

### P78 drug delivery in diabetic *Ins2*^*Akita*^ mice

Kidney tissue samples were obtained from a recently published study that tested physiological effects of P78 on kidney function and pathology [25]. Animals were treated as previously described [25] prior to extracting RNA for RNA-seq analysis. The animal studies were approved by the Penn State University College of Medicine Institutional Animal Care and Use Committee, and performed in strict accordance with the recommendations in the Guide for the Care and Use of Laboratory Animals of the National Institutes of Health. All experiments were conducted using male D2.B6-*Ins2* ^*Akita*^/MatbJ diabetic animals and their wild type (WT) littermate mice (DBA/2 J background), recommended by the Animal Models of Diabetes Complications Consortium (AMDCC) as a model of DN [26, 27]. The diabetic D2.B6-*Ins2* ^*Akita*^ mice develop hyperglycemia at 3 weeks of age and all treatment carried out when the mice were either 6 weeks (3 weeks hyperglycemic exposure; early stage treatment) or 12 weeks (9 weeks hyperglycemic exposure; late stage treatment) of age. Only mice with blood glucose levels > 350 mg/dl (measured using Accu-Chek glucometer, Boehringer Mannheim, Indianapolis, IN) were considered diabetic and used in the study.

The drug tested was P78, a small PEDF active peptide [22, 23], generated by methods previously described [25, 28]. Briefly, P78 peptide at a dose of 0.3 μg/g/day or vehicle (phosphate-buffered saline; PBS) was administered by continuous subcutaneous infusion via the osmotic minipump (no. 2006; Alzet, Durect, Palo Alto, CA), implanted dorsally between the shoulders of the animals as previously described [25, 29–31]. Transcriptome analysis of wild-type and diabetic kidney samples were performed at two stages of diabetes where treatment was initiated at an early stage (6 weeks of age; 3 weeks hyperglycemic) and late stage (12 weeks of age; 9 weeks hyperglycemic). Age, gender, and weight matched diabetic *Ins2Akita* and wild-type non-diabetic controls were used in the study. All animals including wild-type were implanted with an osmotic minipump infused with either vehicle (wt and diabetic controls) or the P78 peptide (diabetic mice). Duration of treatment was 6 weeks with either peptide or vehicle. One group received treatment at the early stage of diabetes (ET, early treatment) at 6 weeks of age and the experiment terminated at 12 weeks of age. Treatment in the second group was initiated at late stage diabetes (LT, late treatment) at 12 weeks of age and terminated at 18 weeks of age. Mice were provided ad lib access to food and water and were euthanized at the end of the experimental period. Kidney samples for RNA extraction were immediately harvested and frozen in liquid nitrogen at the termination of the experiment.

### Tissue samples preparation and RNA isolation

For RNAseq we used 13 kidney tissue samples from wild-type mice, 7 from the diabetic *Ins2* ^*Akita*^ mice, 8 from early P78 treatment of diabetic *Ins2* ^*Akita*^ mice, and 7 from late P78 treatment the diabetic *Ins2* ^*Akita*^ mice [25]. Total RNA was extracted using mirVana kit (Life Technologies) with some modifications. Briefly, a bead mill homogenizer (Bullet Blender, Next Advance) was used to homogenize the tissue using a safe-lock microcentrifuge tube (Eppendorf) and a mass of stainless steel beads (Next Advance, cat# SSB14B) equal to the mass of the tissue. Immediately after two volumes of lysis buffer were added to the tube, samples were mixed in the Bullet Blender for 1 min at a speed of six. Samples were visually inspected to confirm desired homogenization and then incubated at 37 °C for 5 min. The lysis buffer was added up to 0.6 ml, and samples were mixed in the Bullet Blender for 1 min. Optical density values of extracted RNA were measured using NanoDrop (Thermo Scientific) to confirm an A_260_:A_280_ ratio above 1.9. RNA integration number (RIN) was measured using BioAnalyzer (Agilent) RNA 6000 Nano Kit to confirm RIN above 7.

### Library preparation and sequencing

The cDNA libraries were prepared using SureSelect Strand Specific RNA Library Preparation Kit (Agilent) as per the manufacturer’s instructions. Briefly, polyA RNA was purified from 1000 ng of total RNA using oligo (dT) beads. Extracted RNA was subjected to fragmentation, reverse transcription, end repair, 3’-end adenylation, adaptor ligation and subsequent PCR amplification and SPRI bead purification (Beckman Coulter). The unique barcode sequences were incorporated in the adaptors for multiplexed high-throughput sequencing. The final product was assessed for its size distribution and concentration using BioAnalyzer High Sensitivity DNA Kit (Agilent) and Kapa Library Quantification Kit (Kapa Biosystems). 12 libraries were pooled and diluted to 2 nM in EB buffer (Qiagen) and then denatured using the Illumina protocol. The denatured libraries were diluted to 10 pM by pre-chilled hybridization buffer and loaded onto TruSeq SR v3 flow cells on an Illumina HiSeq 2500 and run for 50 cycles using a single-read recipe (TruSeq SBS Kit v3) according to the manufacturer's instructions (Illumina).

### Quality control, mapping and quantification of RNA-Seq reads

Illumina CASAVA pipeline Version 1.8 was used to extract de-multiplexed sequencing reads. FastQC (version 0.11.2) (http://www.bioinformatics.babraham.ac.uk/projects/fastqc/) was used to validate the quality of the raw sequence data. Additional quality filtering used FASTX-Toolkit (http://hannonlab.cshl.edu/fastx_toolkit) using a quality score cutoff of 20. Next, alignment of the filtered reads to the mouse reference genome (mm10) was done using Tophat (version 2.0.9) [32] allowing 2 mismatches. Picard (version1.102) (https://github.com/broadinstitute/picard) was used to assess proportion of mapped bases to coding, UTR, intronic, and intergenic regions, respectively. Picard was used to find coverage across gene body to determine 5’- or 3’- bias. FPKM (Fragments Per Kilobase of Exon Per Million Fragments Mapped) values were calculated using Cufflinks Version 2.0.2 [33] as provided with the Ensembl gene annotation (release 78).

### Differential Gene Expression (DEG) analysis

Only reliably expressed genes were included in the analysis, defined to be those with at least 2 samples with FPKM > = 1. The ComBat function of the sva v3.10.0 R package was used for batch normalization of the FPKM values. The DEGexp function of the DEGseq v1.18.0 R package [34] was used to identify differentially expressed genes (DEG) between diabetes and control (DvC), treatment and diabetes (TvD), early treatment and diabetes (ETvD) and late treatment and diabetes (LTvD), using the Likelihood Ratio Test method. Significantly DEG were defined to be those with q-value < 0.05 calculated by the Storey et al. 2003 method.

### Visualization

The batch adjusted RNA-seq FPKM values of the reliably expressed genes were averaged for samples in each category: C, D, ET and LT. A heatmap of the Z scaled resulting FPKM values was generated using the heatmap.2 function of the gplots R package, with parameters “average” for clustering method and 1-correlation for distance. The Z scaling was performed with the genefilter v1.46.1 R package. A volcano plot was generated of the –log10(*p*-value) vs. log2(normalized FC) of the DvC and TvD DEG analyses, with points with q < 0.05 and abs(log2(normalized FC)) > = 1 (i.e. a FC threshold of 2) colored red for DvC and green for TvD. Labeled genes have a q-value < 0.05 and abs(log2(normalized FC)) > = 2 (i.e. a FC threshold of 4). Quad Venn diagrams showing the number of significant DEG genes and protein coding subsets for various comparisons were generated using the VennDiagram v1.6.9 R package.

### Functional analysis

The Tox Analysis function of Ingenuity Pathway Analysis (IPA) (QIAGEN, California) was used to create gene sets based on biological processes and toxicological responses to xenobiotic insult using the mammal filter (human, rat, mouse) and the following tissues in turn: adipose, brain, heart, kidney, liver, retina and all tissues. IPA Build 2015-03-23.

### Statistical analysis

Statistical tools embedded in the previous instruments and statistical packages were applied. Significant DEG were defined to be those with q-value <0.05 calculated by the Storey et al. 2003 method.

### RT-PCR

Total mRNA from treated, non-treated, and wt kidney samples was extracted for PCR using the RNAeasy Mini Kit (Qiagen) First-strand cDNA of isolated mRNA was synthesized using Superscript First Strand cDNA Synthesis reagents (Invitrogen). Gene-specific primers were designed using NCBI Primer-Blast primer design tool (Primer3web version 4.0.0) and primer information listed in the table below. Reverse transcription (RT)-PCR was carried out using 80 ng cDNA from pooled samples, PCR master mix reagents (Invitrogen), annealing temperature of 58 °C, and 35 amplification cycles. PCR products were resolved by 1% agarose gel electrophoresis. Quantitative RT-PCR (qrt-PCR) was also performed using 20 ng cDNA and these primers to cross-validate, quantitate, and confirm gene expression changes and trends. For qrt-PCR, a two-step amplifying protocol was used with iQ SYBR Green Supermix solution (Bio-Rad). C_t_ (threshold cycle) was used to determine gene expression levels.

**Table:** 

Genes	ENSEMBL #	Primer sequence (5’-3’)	Amplicon size (bp)
Nhej1	ENSMUSG00000026162	F: 5’ CCAAGCACGGTTATGCCTTG 3’ R: 5’ CAGGCTCACACCCATCAGAG 3’	344
Ept1	ENSMUSG00000075703	F: 5’ TTCAGCCAGAGATGCCAGTG 3’ R: 5’ CAGGCACCCAATCCTAGCAA 3’	274
Cyp4a14	ENSMUSG00000028715	F: 5’ TTGCCAGAATGGAGGATAGGAA 3’ R: 5’ TGGAGCGTCCATCTGGGAAG 3’	324
Ugt1a2	ENSMUSG00000090171	F: 5’ GCCCCTTCGAGGAATCTCAG 3’ R: 5’AGGTCTTCTCAATGTCGCTCAG 3’	278
KDM4B	ENSMUSG00000024201	F: 5’ TCCACCAACACCCCTCAATG 3’ R: 5’ AAGGGGTTGTTCCCTGTGAG 3”	218
Selm	ENSMUSG00000075702	F: 5’ CACCAACTACCGACCGGATT 3’ R: 5’ TCCTGTACCAGCGCATTGAT 3’	261
Mamdc4	ENSMUSG00000026941	F: 5’ GCTCCTGGGCACTTCCTATC 3’ R: 5’ GAGTCACATTGTCCACCCCA 3’	334

## Results

### Global transcriptome changes

Results obtained from DEGseq analysis applied on RNA-seq FPKM (Fragments per kilobase of exon per million fragments mapped) showed significant differences in coding and non-coding genes expressed in the kidney among control (C, wild-type), diabetic (D), early (ET), and late (LT) P78 treatment in the Ins2Akita mouse model of diabetic nephropathy (DN). Below we provide results for protein coding and non-coding genes that were regulated in the diabetic kidney relative to P78 treated and wt controls: The comparisons are (1) diabetic relative to wild type (DvC), (2) P78 treated relative to non-treated diabetic (TvD), (3) diabetic returned to normal levels by treatment (early and late stage treatment), and (4) P78 early stage treatment (ET) compared to late stage treatment (LT). Early (ET) and late (LT) P78 treatments are grouped in the TvD analyses to determine all gene targets of the treatment whether given at early or late stages of diabetes. These are later separated in the study to determine those targets that were unique to ET and those unique to LT.

### Global expression changes

**Fig. 1:**
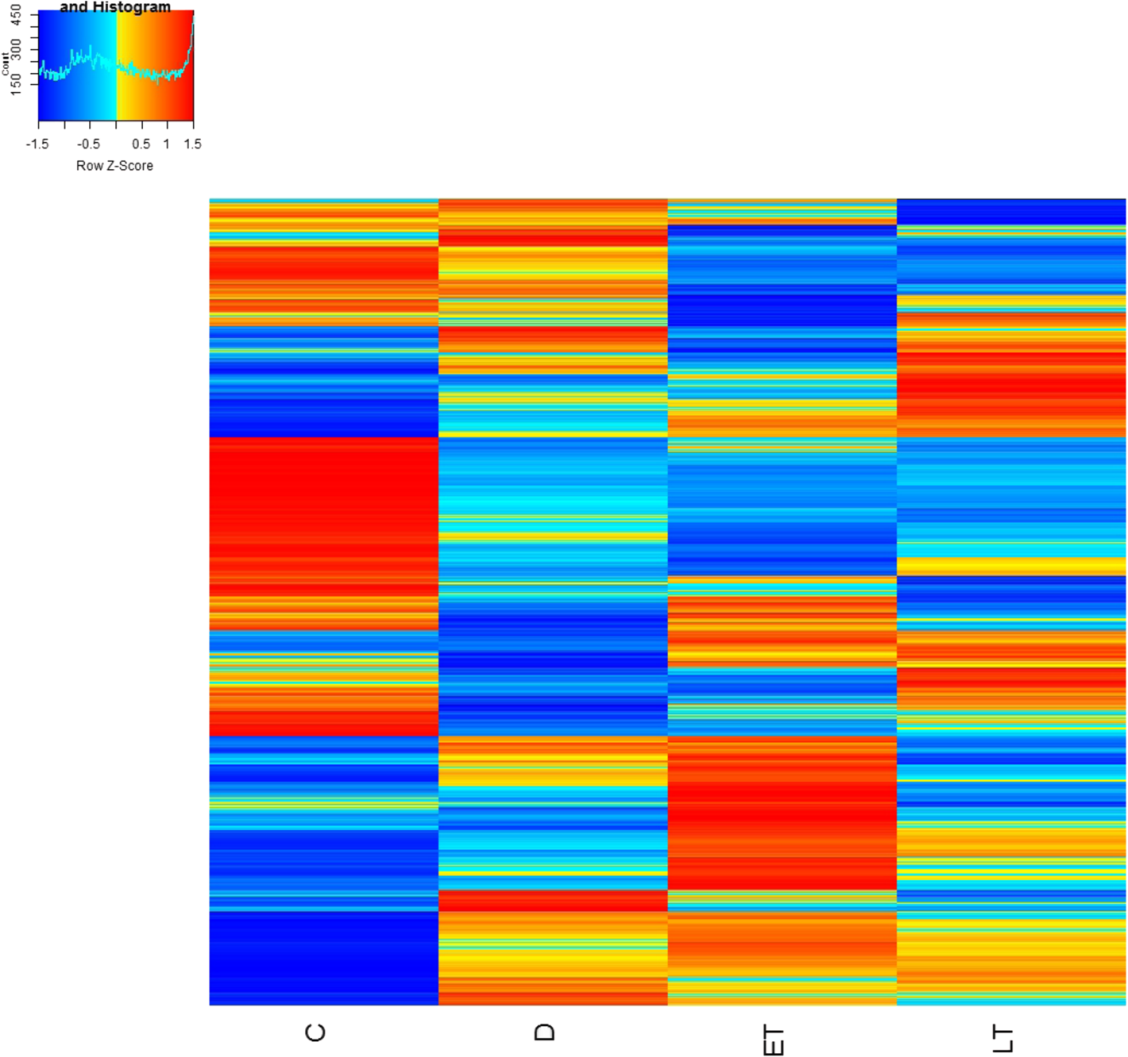
Heatmap of Z scaled RNA-seq FPKM (Fragments per kilobase of exon per million fragments mapped) values of 14,316 reliably expressed transcripts in kidney samples of the *Ins2* ^*Akita*^ mouse model of diabetic nephropathy (DN). FPKM values were averaged for samples in each category (C: control, D: diabetic, ET: early P78 treatment, LT: late P78 treatment) (*n* = 7-13)

**Fig. 2:**
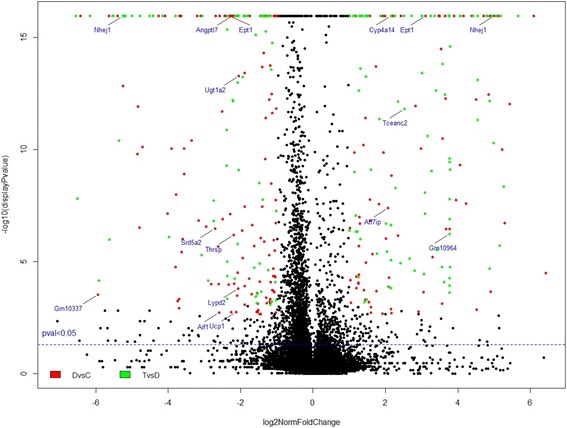
Volcano Plot showing –log10(*p*-value) vs log2(normalized fold change (FC)) of RNA-seq FPKM (Fragments per kilobase of exon per million fragments mapped) for all transcripts (protein-coding and non-coding) differentially expressed genes (DEG) in kidney samples of *Ins2* ^*Akita*^ mouse model of diabetic nephropathy. Distribution of genes with respect to significance (y axis) versus fold changes (x axis) is shown. Genes of interest outside the midline where absolute normalized FC > =+/-4 (q < 0.05) are labeled as examples to show changes in expression levels in diabetic (DvC, Red) or P78 treated diabetic mice (TvD, Green) relative to controls. Horizontal dashed line represents expression value *p* = 0.05; above line *p* < 0.05; below line: *p* > 0.05. Genes outside the midline have FC > 1

The heatmap in Fig. 1 and volcano plot in Fig. 2 show global transcriptome changes in control, diabetic, and P78 early and late treatments in kidney tissues of the *Ins2* ^*Akita*^ mouse model of diabetic nephropathy. 43,168 RNA transcripts were identified in the kidney. 25,254 had an FPKM value > 0 and 14,316 were considered reliably expressed, defined to be those with FPKM > =1. Of the 14,316 reliably expressed sequences, 12,328 were protein coding genes and 1,988 non-coding RNA transcripts.

The distribution of all genes (protein-coding and non-coding) with respect to significance (y axis) versus fold changes (x axis) is shown in the scatter plot in Fig. 2 (–log10(*p*-value) vs log2(normalized fold change (FC)). Genes of interest are outside the midline where absolute normalized FC > =(+/-)4 are colored red for DvC (diabetic versus wt controls) and green for TvD (early and late stage P78-treated diabetic mice versus diabetic controls). A few genes including Nhej1, Ept1, Cyp4a14, and Ugt1a2, which are significantly regulated by diabetes and treatment with an absolute normalized FC > =(+/-)4, are labeled as examples to show their location and relationship to other points in the plot and to the midline. Many protein-coding and non-coding genes showed small changes in their expression levels in either direction but only those that met our selection criteria of FC > =(+/-)1.5, q < 0.05 were of interest in this study and rank-ordered in all tables according to fold changes in either direction.

### All transcripts regulated by diabetes and P78

**Fig. 3:**
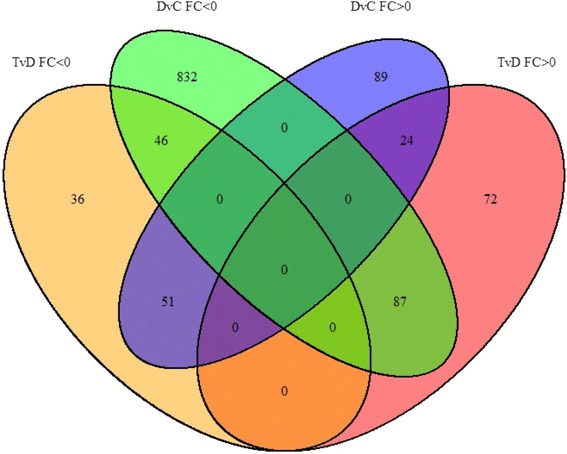
The Venn Diagram shows RNA-seq data for all RNA biotypes (protein-coding and non-coding) that were regulated by diabetes compared to control (DvC) and by P78 treated compared to non-treated diabetic controls (TvD). FC: fold changes. Blue: DvC - positive FC (FC > 0). Green: DvC - negative FC (FC < 0). Pink: TvD - positive FC (FC > 0). Yellow: TvD - negative FC (FC < 0). Changes in expression levels were seen in 1237 transcripts of which 208 were regulated by both diabetes and treatment. 1129 transcripts were regulated in DN, 316 by treatment (early and late), and 138 whose expression levels were altered in DN were reversed by treatment towards control levels (q < 0.05)

Gene expression differences in the kidney between the diabetic compared to control (DvC) and P78 treatment compared to diabetic (TvD, early and late) groups are shown in the Venn diagram in Fig. 3 and in Table 1. These represent all protein-coding and non-coding RNA transcripts that showed statistically significant differential expression (DEG) (q < 0.05). Changes in expression levels were seen in 1237 transcripts in the kidney (a total of all numbers in the Venn diagram). 208 (46 + 51 + 87 + 24) of these were regulated by both diabetes and treatment. 1,129 RNA transcripts (coding plus non-coding) were regulated by diabetes with log2 fold changes (FC) ranging between -86.2 and +86.0 (q < 0.05) (Table 1). 164 were upregulated and 965 downregulated by diabetes. P78 treatment (early and late; TvD group) altered expression of 316 transcripts with FC between -93.3 and +50.9 (q < 0.05). Of these, 183 were upregulated and 133 downregulated. A total of 138/1129 (12.2%) transcripts whose levels were either increased (51 transcripts) or decreased (87 transcripts) by diabetes were reversed in the direction of their expression to normal levels by P78 treatment (Table 1). While the regulation of most transcripts by diabetes were not reversed by treatment, the effects of P78 in reducing pathology and progression of DN that we have previously shown [24, 25] may be due, in part, to reversing diabetes-induced expression changes in this subset (138) of coding and non-coding RNA transcripts.

**Table 1:** Number of all transcripts (coding and non-coding) that were significantly regulated in the kidney by diabetes (D), P78 treatment (T, early and late treatment), and those with expression changes in D that were reversed by T in the *Ins2* ^*Akita*^ mouse model of diabetic nephropathy (DN) (q < 0.05)

Group	# of Genes
Upregulated in D	164
Downregulated in D	965
Upregulated with T	183
Downregulated with T	133
Upregulated in D, reversed by T	51
Downregulated in D, reversed by T	87

### Protein-coding genes (mRNA) regulated by diabetes and P78

**Fig. 4:**
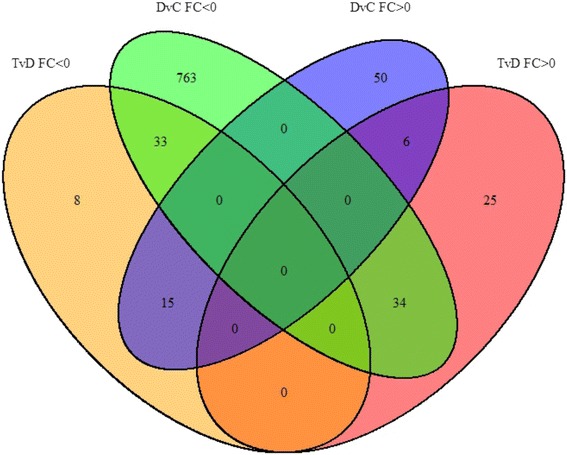
The Venn diagram shows RNAseq data for only protein-coding genes that are regulated by diabetes or P78 treatment. Expression changes were detected in 934 protein-coding genes with 88 regulated by both diabetes and treatment. 901 coding genes were regulated by diabetes, 121 by P78 treatment (early and late) and 49 coding genes with changes induced by diabetes were reversed by treatment toward control levels of expression (q < 0.05)

Figure 4 and Table 2 show the number of protein coding genes (mRNA) regulated by diabetes and P78. Changes in gene expression occurred in 934 protein-coding genes (all numbers in the Venn diagram) in the kidney of the *Ins2* ^*Akita*^ mouse model of DN, with 88 of these (33 + 15 + 34 + 6) regulated by both diabetes and treatment. Diabetes altered expression of 901 genes (q < 0.05), of which 71 were upregulated and 830 downregulated by the disease (Table 2). P78 (early and late treatment) modulated expression of 121 coding genes with 65 of these showing increased and 56 showing decreased expression levels. P78 treatment reversed direction of expression of 49/901 (5.44%) of the protein coding genes regulated in diabetic kidneys towards normal levels (Table 2).

**Table 2:** Number of protein-coding (mRNA) genes that were significantly regulated in the kidney by diabetes and by P78 treatment compared to non-diabetic controls, and those with expression changes in diabetes that were reversed towards normal levels by P78 treatment in the *Ins2* ^*Akita*^ mice with FC > (+/-)1 (q < 0.05)

Group	# of Genes
Upregulated in D	71
Downregulated in D	830
Upregulated with T	65
Downregulated with T	56
Upregulated in D, reversed by T	15
Downregulated in D, reversed by T	34

Only protein-coding genes with the highest fold changes induced by diabetes or P78 are shown in Table 3A-C. These represent all differentially expressed genes with absolute FC > =(+/-)1.5 (q < 0.05) and are rank-ordered by FC in either direction. Table 3A lists protein-coding genes that were regulated by diabetes of which Nhej1 (+32.04), Ept1 (+8.6), Srd5a2 (-6.55), Aif1 (-6.05), Angptl7 (-4.71), Thrsp (-4.57), Cyp4a14 (+4.4), Ucp1 (-4.35), Atf7ip (+4.21), Lypd2 (-4.2), and Ugt1a2 (-4.13) had FC > (+/-)4.0 (q < 0.05).

**Table 3:** Differentially expressed protein coding genes (mRNA) showing the highest fold changes (in either direction) in kidney samples of the *Ins2* ^*Akita*^ mice in the diabetic (D) and P78 treatment (T) groups

Increased		Decreased	
Gene	Fold change	Gene	Fold change
A			
Nhej1	32.04	Srd5a2	−6.55
Ept1	8.6	Aif1	−6.05
Cyp4a14	4.44	Angptl7	−4.71
A@7ip	4.21	Thrsp	−4.57
Foxn3	2.92	Ucp1	−4.35
Txnrd1	2.76	Lypd2	−4.2
Gldc	2.44	Ugt1a2	−4.13
Cyp4a12b	2.4	Ly6e	−3.77
Sulf2	2.37	Scd1	−3.7
Kdm4b	2.28	Inmt	−3.42
Suco	2.28	Ang	−3.24
Cyp4a10	2.27	Cyp2d12	−2.94
Aldh1a7	2.21	Anxa13	−2.92
Slc18b1	2.04	Car9	−2.89
Reep6	1.97	Mamdc4	−2.85
Gsta2	1.95	Cox8b	−2.77
Zbtb18	1.94	Tmem86a	−2.64
Slc38a3	1.93	Il34	−2.57
Akr1d1	1.91	Ramp3	−2.51
Etf1	1.86	Acsm3	−2.5
ApoB	1.85	Gusb	−2.48
Erlin1	1.8	Hsd17b11	−2.43
Pklr	1.78	Cyp4b1	−2.35
Folh1	1.77	Rarres2	−2.32
Cfd	1.71	Lipo1	−2.32
Huwe1	1.7	Nudt19	−2.28
Itgb6	1.69	Cck	−2.28
Cyp4a31	1.65	H2-Ab1	−2.27
Mtus1	1.65	Tmem37	−2.22
Selm	1.64	Eri2	−2.2
Ccng1	1.64	C1qtnf3	−2.18
C3	1.61	Rhod	−2.18
Enpep	1.6	Stab1	−2.14
Mgst1	1.56	Hsd11b1	−2.13
B			
Tceanc2	5.76	Nhej1	−37.94
Ugt1a2	3.03	Ept1	−4.45
Mamdc4	2.81	Tmsb15l	−3
Plk3	2.68	Kdm4b	−2.8
Lars2	2.57	Selm	−2.47
Sgk1	1.76	Cyp4a14	−2.22
Tmem252	1.75	Cyp24a1	−2.02
Adck5	1.72	Alox5ap	−1.92
Psmd13	1.68	Etf1	−1.71
Chic2	1.67	Hba-a1	−1.54
Slc25a25	1.61	Odc1	−1.52
Vps39	1.61	Hbb‐bs	−1.51
Hacl1	1.6		
Gcn1l1	1.57		
Hpd	1.57		
Acnat2	1.54		
Fkbp5	1.5		
C			
Gene	DvC	TvD	
Nhej1	32.04	−37.94	
Ept1	8.6	−4.45	
Cyp4a14	4.44	−2.22	
Ugt1a2	−4.13	3.03	
Mamdc4	−2.85	2.81	
Kdm4b	2.29	−2.8	
Etf1	1.86	−1.71	
Chic2	−1.85	1.67	
Tmem252	−1.67	1.75	
Selm	1.64	−2.47	
Fkbp5	−1.6	1.5	
Hpd	−1.58	1.57	

A. All protein-coding genes with increased or decreased expression levels by FC > (+/-)1.5 (q < 0.05) in diabetic relative to wt control B. All coding genes upregulated or downregulated by P78 treatment with FC > =(+/-)1.5 (q < 0.05) relative to diabetic controls. C. All coding genes with FC > (+/-)1.5 (q < 0.05) that were reverted in their expression towards normal levels by P78 treatment (TvD, early and late treatment). Genes are rank ordered by FC (positive or negative values)

Table 3B lists genes (FC > =(+/-)1.5 in diabetic animals that showed changes in expression by P78 treatment (early and late) of which the top 5 include Nhej1 (-37.94), Tceanc2 (+5.76), Ept1 (-4.45), Ugt1a2 (+3.03), and Tmsb15l (-3.0) with FC > (+/-)3.0 (q < 0.05).

### P78 Treatment normalized gene expression altered by diabetes

From this analysis (Table 3A and 3B), we identified a small set of genes where the changes in diabetes were substantially (~50%) or completely reversed by P78 treatment. These are listed in Table 3C and rank-ordered by FC > =(+/-)1.5 (q < 0.05). Of these, non-homologous end-joining factor 1 (Nhej1) had the highest absolute fold change in expression with both diabetes and treatment. Diabetes induced expression levels of Nhej1 by +32.04 while P78 complete reversed expression by -37.94 fold changes. Treatment also completely reversed diabetes-induced expression changes of Mamdc4, Kdm4b, Tmem252, Selm, and Hpd while others were returned in the direction of normal levels from their diabetes-induced state by ~50% (Table 3C).

**Fig. 5:**
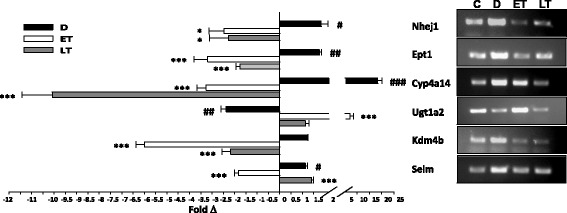
PCR of pooled kidney samples (*n* = 7-13) from Control (C; wt), Diabetes (D), P78 treatment early stage diabetes (ET) and P78 treatment late stage diabetes (LT). QRT-PCR (*left*) and RT-PCR (*righ*t) analyses confirm the trend of the rRNAseq data for regulation of a group of genes that were modulated with FC > =(+/-)2 by both diabetes and P78 treatment. Wt controls (C, not indicated as bars in the graph) are set to 1. (DvC: #*p* ≤ 0.05; ##*p* ≤ 0.01; ###*p* ≤ 0.001; ETvD or LTvD: *p ≤ 0.05; **p ≤ 0.01; ***p ≤ 0.001)

Regulation of genes with FC > =(+/-)2.0 (q < 0.05) by diabetes, or treatment (P78 early (ET), and P78 late (LT)) (Table 3C) was subsequently cross-validated for trend in expression by RT-PCR and QRT-PCR (Fig. 5) with the exception of Mamdc4, whose expression level was below the detection threshold using these PCR conditions. While regulation trends remain the same, some differences in levels of gene regulation between the RNAseq and PCR data were noted, which were possible due to primer specificity, alternatively spliced forms of the genes, RNA integrity, and/or PCR conditions.

This small subset of genes regulated in opposite directions by both diabetes and P78, a treatment previously shown to reduce DN pathology [24, 25] may be critically involved in diabetes-induced mechanisms that control the development of the disease.

### Transcripts regulated by early and late stage P78 treatment

Since treatments were given at early and late stage diabetes, we sorted the data to identify genes that were regulated by early treatment and those regulated by late treatment (Table 4). Several transcripts were unique targets of P78 in early and in late stages of diabetes but many were common targets of P78 regardless of the stage of diabetes (Table 4A and 4B) in which case treatment completely reversed diabetes-induced regulation. Nhej1, for example showed regulation from FC = +32.0 in the diabetic kidney (Table 3A) to -38.55 and -37.33, for early and late treatments, respectively (Table 4A, 4B). Nhej1, a DNA repair gene, showed the highest fold changes of the protein coding genes regulated by P78 treatments (ET, LT) in both stages of diabetes. We identified sixteen protein-coding genes that were targets of P78 that were significant and reliably altered in expression levels regardless of the diabetic stage when treatment was given (Table 4B). These represented ~50% similarity in gene expression changes by treatment at both early and late stages of diabetes. With the exception of C3, which showed increased expression (+2.06) with early treatment and change in expression in the opposite direction (-1.41) with late treatment, the other 15 genes were regulated in the same direction by P78 and with similar FC in both diabetic stages treated (Table 4B). Many differences in genes showing expression changes were also observed between the early and late stage diabetes treatments. For example in early diabetes treatment increased levels of Ugt1a2 (+5.41 FC), S100a9 (+4.23 FC), Plk3 (+3.82 FC), and Lcn2 (+3.38 FC) and decreased levels of Cyp24a1 (-2.51 FC) and Tfpi2 (-1.76 FC), genes whose expression changes were not evident with late treatment using our cut off criteria. Others that showed changes in gene expression with late stage diabetes treatment including Alox5ap (-5.18 FC), Etf1 (-2.48 FC), Psmd13 (+1.98 FC), and Adck5 (+1.89 FC) did not show changes with early stage treatment.

**Table 4:** Protein coding genes differentially expressed with the highest positive or negative FC in kidney samples of the *Ins2* ^*Akita*^ diabetic mice

A			
ET vs D		LT vs D	
Gene	Fold change	Gene	Fold change
Nhej1	−38.55	Nhej1	−37.33
Tceanc2	6.03	Ept1	−7.87
Ugt1a2	5.41	Tceanc2	5.48
S100a9	4.23	Alox5ap	−5.18
Lars2	3.99	Cyp4a14	−4.74
Plk3	3.82	Mamdc4	3.78
Lcn2	3.38	Kdm4b	−2.82
Ept1	−3.12	Etf1	−2.48
Kdm4b	−2.79	Selm	−2.37
Selm	−2.58	Psmd13	1.98
Cyp24a1	−2.51	Adck5	1.89
Cyp27b1	2.33	Ugt1a6a	1.76
Arhgap27	2.22	Ndufaf1	1.71
Sgk1	2.22	Vps39	1.64
Fga	2.13	Rfxap	1.62
Tmem252	2.09	Chic2	1.61
C3	2.06	Erlin1	−1.61
Angptl4	2.05	Hacl1	1.59
Slc25a25	2.04	Hpd	1.56
Ddit4	2.03	Pnrc2	1.52
Ddit3	1.92		
Fkbp5	1.81		
Acnat2	1.79		
Aff4	1.79		
Alas1	1.78		
Tfpi2	−1.76		
Tsc22d1	1.76		
Chic2	1.72		
B			
ET, LT overlapping genes			
Gene	ETvD	LTvD	
Nhej1	−38.55	−37.33	
Ept1	−3.12	−7.87	
Tceanc2	6.03	5.48	
Cyp4a14	−1.46	−4.74	
Kdm4b	−2.79	−2.82	
Selm	−2.58	−2.37	
Etf1	−1.31	−2.48	
C3	2.06	−1.41	
Psmd13	1.39	1.98	
Chic2	1.72	1.61	
Hba‐a1	−1.65	−1.44	
Vps39	1.57	1.64	
Hacl1	1.62	1.59	
Hbb‐bs	−1.59	−1.43	
Odc1	−1.58	−1.46	
Hpd	1.57	1.56	

The tables show only those genes that were significant and reliably expressed with FC > =(+/-)1.72 (ET), FC > =(+/-)1.52 (LT) (q < 0.05) and those that were regulated by both treatments (overlapping).These were rank-ordered by highest numerical +/- FC values. A. P78 treatment administered at early (ET, early treatment) or late (LT, late treatment) stage diabetes relative to diabetic controls. 28 genes were regulated by treatment with P78 at the early stage of diabetes and 20 by treatment at the late stage of diabetes. B. The table shows all protein-coding genes that were regulated by P78 in the same direction (except C3) at both stages of diabetes which represented ~50% of the genes

### Non-coding small RNAs (miRNA)

Diabetes and P78 also regulated a group of small non-coding RNA precursor biotypes, with yet unknown or speculative function but are generally believed to control expression and biological function of coding genes. Twenty nine of these with the greatest expression changes (FC > (+/-)5.5 (q < 0.05) were identified in the diseased kidney compared to wild-type controls (Table 5A) and include miRNA, snRNA, snoRNA, and pseudogene biotypes. miRNAs highly regulated in DN with FC > =(+/-)20 (q < 0.05) include Mir1247 (-86.22), Mir142b (-42.04), Mir1905 (+29.52), Mir196a-1 (+22.61), Mir27a (+20.80) (Table 5A). From this analysis, we also identified a set of small RNAs, categorized as miRNA by Gencode, that was highly regulated by diabetes (DvC) and whose expression levels were completely or partially reversed by P78 treatment (TvD). These are rank ordered by FC (up/down) in Table 5B. Of interest in the development and treatment of DN are those miRNA regulated by diabetes whose expression levels were complete reversed by P78 treatment including Gm24083, Gm25953, and Gm25535, GM25872, Mir1898, Gm25361, Mir7036, Mir7001, and Mir3098. Others including Mir1247 (-86.22) and Mir142b (-42.04) where expression changes in diabetes were not significantly returned to normal levels by treatment. While the functions of many of these sequences are still being elucidated, they represent a class of RNAs that may be useful in understanding the DN pathobiology and in developing treatments for the disease.

**Table 5:** Differentially expressed precursor miRNAs including Gms assigned as miRNA by Gencode with highest FC in kidney samples of the *Ins2* ^*Akita*^ mice

A		B		
Gene	Fold change	Gene	DvC	TvD
Mir1247	−86.22	Gm24083	43.15	−45.45
Gm24083	43.15	Gm25953	34.53	−36.37
Mir142b	−42.04	Mir1905	29.52	−5.19
Gm25953	34.53	Gm25535	−28.56	23.41
Mir1905	29.52	Gm27903	−26.25	11.75
Gm25535	−28.56	Mir196a‐1	22.61	−2.35
Gm27903	−26.25	Gm25738	−17.06	10.57
Gm23251	22.85	Gm25872	−16.27	23.35
Mir196a‐1	22.61	Gm28010	15.43	−3.08
Mir27a	20.80	Mir6991	14.82	−2.65
Mirlet7b	18.64	Mir6364	−12.80	8.06
Gm25738	−17.06	Gm27684	12.51	−1.86
Mir6386	16.74	Mir328	−11.78	8.56
Gm25872	−16.27	Mir1898	−9.23	9.49
Gm28010	15.43	Gm25361	−8.57	10.18
Mir6991	14.82	Gm23134	−6.45	1.80
Mir6981	13.64	Mir7036	−5.33	16.87
Mir6364	−12.80	Mir7001	−4.92	4.78
Gm27684	12.51	Mir3098	4.61	−3.51
Gm24515	11.93	Gm27768	−3.70	4.45
Mir328	−11.78	Gm25233	−3.70	4.45
Gm25297	11.09	Gm22734	−3.70	4.45
Mir1898	−9.23	Gm26043	2.96	−8.34
Gm25361	−8.57	Gm22721	−2.76	2.17
Gm27920	7.86	Gm25640	−2.43	6.59
Gm27512	7.53	Mir6363	−2.08	2.27
Gm28033	7.13	Gm25190	−1.93	2.33
Gm23134	−6.45	Mir27b	−1.84	2.01
Gm23268	−5.97	Mir3082	−1.66	1.57

A. miRNAs regulated by diabetes with FC > =(+/-)5.97 (q < 0.05) relative to control.Genes are listed by highest numerical FC values (positive or negative), B. miRNAs with expression levels altered by diabetes that were reversed by P78 treatment. Genes are listed by highest numerical FC values (FC > =+/-1.66, q < 0.05) in DvC (first column) and the relative regulation values of these same genes after treatment (TvD) given in the second column

### Biological function - pathways Ingenuity Pathway Analysis (IPA)

**Fig. 6:**
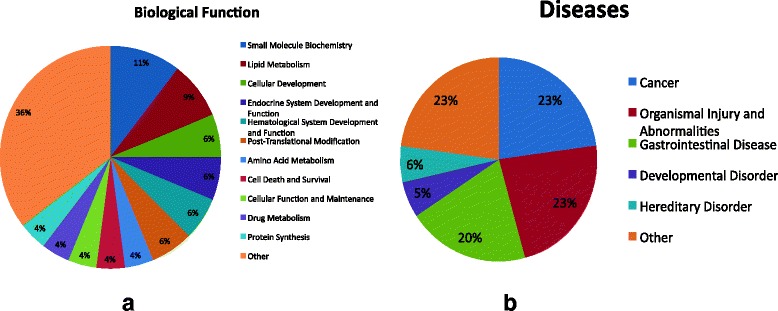
**a**. Biological functions associated with 12 target genes (Table 3C) regulated by diabetes that were completely reversed in their expression to control levels by P78 treatment, **b**. Disease relatedness of these genes are shown with percentage indicating number of genes in each group

IPA analysis shows that protein-coding genes regulated by diabetes and returned to near normal levels by treatment (Table 3C) are associated with key biological functions including lipid metabolism, post translational modification, endocrine and hematological functions, cell death and survival, and protein synthesis, all important processes associated with the disease (Fig. 6a). Many of these are also associated with other pathologies including organismal injury and abnormalities, cancer, developmental and hereditary disorders (Fig. 6b)

**Fig. 7:**
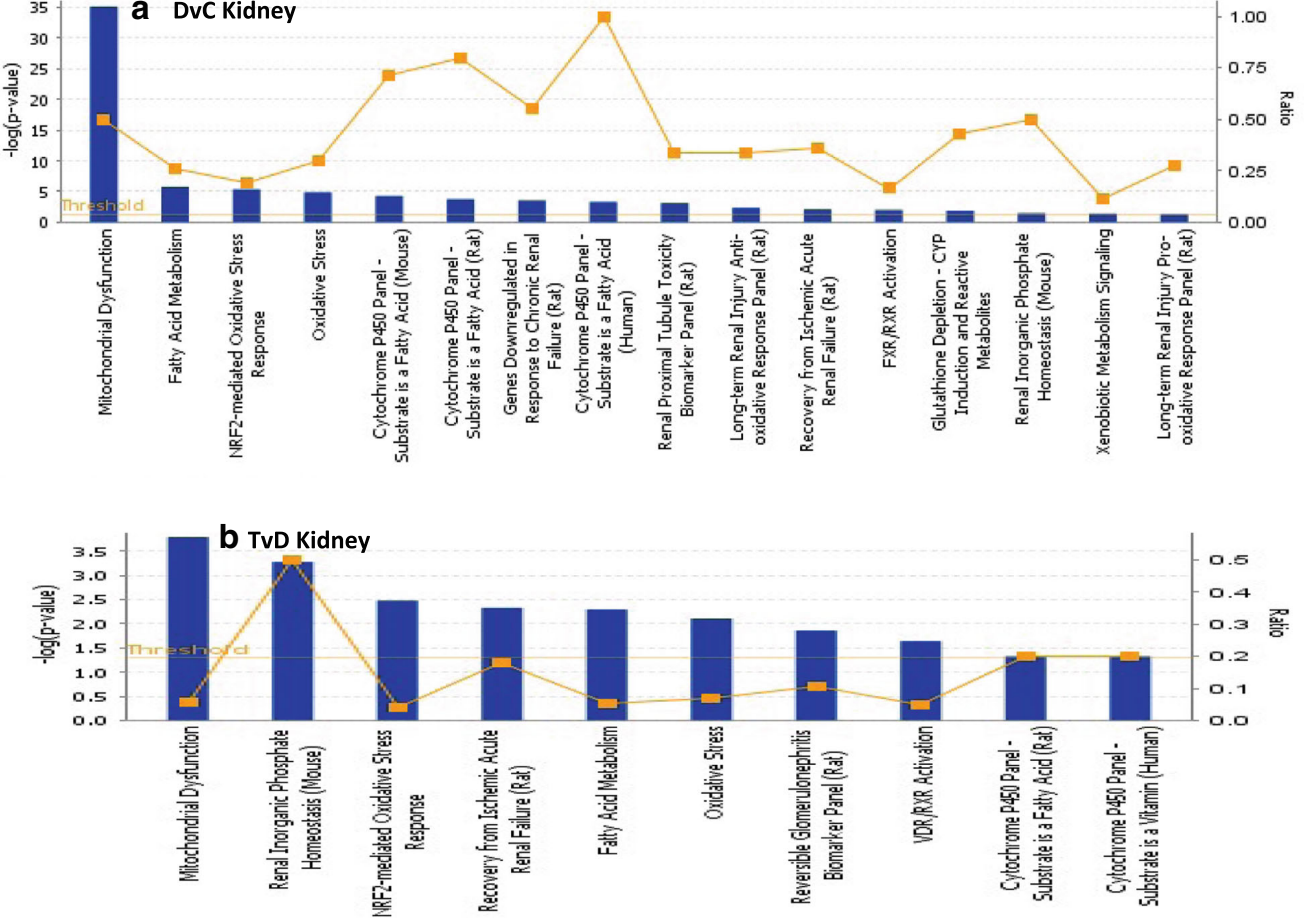
Canonical pathways identified by Ingenuity pathway analysis (IPA) gene ontology algorithms for all significant and reliable DEG in the dataset. **a**. DvC: diabetic *Ins2* ^*Akita*^ mouse kidney samples. **b**. TvD: kidney samples obtained from *Ins2* ^*Akita*^ mice after 6 weeks of continuous P78 infusion. Canonical pathways are displayed along the x-axis and the –log(*p*-value), calculated by Fisher’s exact test right-tailed, on the y-axis. Threshold set to 1.5 indicates the minimum significance level (–log(*p*-value). Height of the bars represents significance (probability that DEG in the dataset are associated with a canonical pathway) with taller bars representing more significant associations. The ratio (orange points) indicates the number of DEG (FC > 1.5; q < 0.05) in the diabetes and treatment datasets that map to a given canonical pathway divided by the total number of genes in that pathway within the IPA reference list

**Fig. 8:**
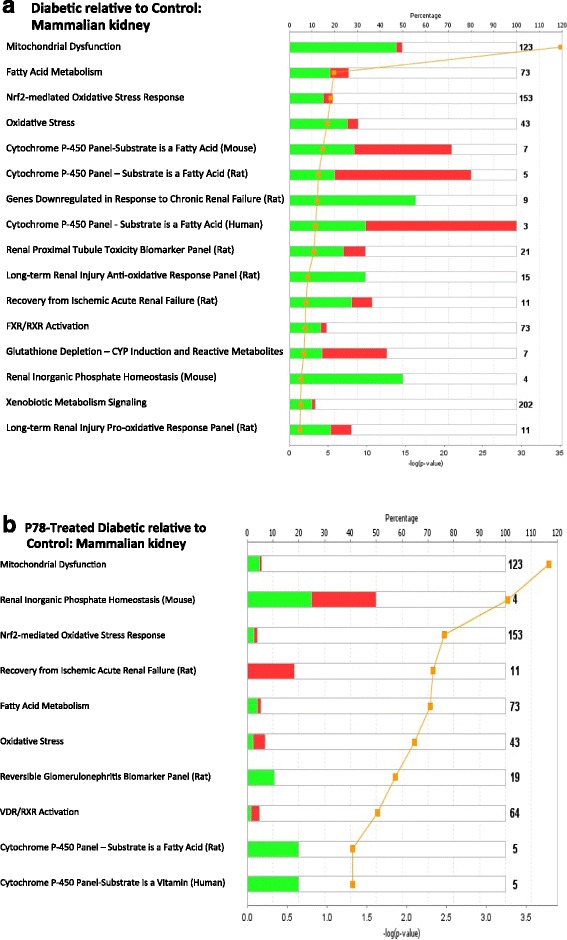
Regulation profile of genes in the canonical pathways most significantly associated with DEG in our RNAseq dataset. **a**. Diabetic *Ins2* ^*Akita*^ kidney samples relative to control. **b**. P78-treated diabetic *Ins2* ^*Akita*^ kidney samples relative to control. The stacked bars show the percentage of genes (y-axis) distributed according to the direction of gene regulation changes in each canonical pathway (x-axis). Genes that were significantly up-regulated in our dataset are shown in red, those that were down-regulated in green, and those found in the IPA reference gene set that had no overlap with our dataset represented as open bars. The numerical value at the right of each canonical pathway bar indicates the total number of genes present in the IPA Knowledge base for that pathway. The orange line represents the significance of the directional change (–log(*p*-value) calculated by adjusting the right-tailed Fisher’s Exact *t*-test *p*-values using the Benjamini-Hochberg method

IPA gene ontology algorithms and KnowledgeBase mining identified several important canonical pathways enriched for the differentially expressed genes (DEG) in our dataset. DEG fell into one of several functionally relevant canonical pathways associated with diabetes and kidney pathology. Figure 7 shows the top pathways represented with FC > =(+/-)1.5 (q > 0.05) and Fig. 8, the percentage of genes regulated relative to those present in the IPA knowledgebase that are assigned to a given pathway and the direction of their regulation (red: upregulated; Green: downregulated) for diabetic (DvC) and P78 treatment (TvD) groups (relative to wt controls). The total number of genes assigned to a pathway in the IPA KnowledgeBase is given to the right of each bar. The open area of each bar represents genes in the pathway that are not represented in our dataset. Pathways were rank-ordered by IPA base on the significance of the directional change (–log(*p*-value) and are limited to those enriched in IPA KnowlegeBase.

The graph in Fig. 8a shows a striking trend in diabetes-induced DEGs with mitochondrial dysfunction in the diabetic group relative to wild type controls. The largest functional cluster of genes modulated by diabetes was represented in the mitochodrial dysfunction canonical pathway. Approximately 50% of the 123 genes assigned to this pathway were differentially expressed by diabetes and ~48% of these were downregulated. The overall trend observed from this analysis was that diabetes decreased expression of more kidney genes clustered in these pathways (12/16) when compared to controls. In three of these pathways associated with chronic renal injury, renal failure, and renal inorganic phosphate homeostasis, all genes were downregulated by diabetes that were present in our kidney dataset.

P78 targeted some of the same pathways as diabetes but fewer genes showed altered expression levels after treatment relative to controls (Fig. 8b). For example, ~50% of the genes in the mitochondrial pathway showed changes in expression in the diabetes group relative to controls while only ~5% showed altered expression in the treated diabetes group relative to controls. Genes in the P78 treated diabetic mice represented in the cytochrome *P*-450 and reversible glomerulonephritis associated pathways were all downregulated compared to those in the same pathway for non-treated mice. While those assigned to the recovery from ischemic acute renal failure pathway showed increased levels of expression after treatment compared to the non-treated diabetic mice. Since we previously showed that P78 significantly reduced physiological and pathological changes in diabetic nephropathy [24, 25] these target pathways and their associated genes may give mechanistic clues to the disease.

## Discussion

In this study we identified a set of transcriptome changes in the kidney of the *Ins2* ^*Akita*^ mouse model of diabetic nephropathy (DN) with and without PEDF-P78 peptide treatment. Kidney samples were obtained from our recently published studies in which we showed that continuous infusion of P78 reduced albuminuria, blood urea nitrogen, and progression of DN [24, 25]. The results from this study provides a panel of biomarkers that shed light into the DN pathology and validate the use of P78 as a therapeutic approach for renal injury.

Using an RNAseq approach [35], we identified a panel of genes dysregulated in DN. Genes showing the greatest decrease in expression in DN include Srd5a2 (5 alpha reductase), Aif1 (Allograft inflammatory factor 1), a protein that modulate insulin secretion in prediabetic rats [36], Angptl7 (angiopoietin-related protein 7), Thrsp (thyroid hormone-inducible hepatic protein), and Ucp1 (thermogenin), found in the mitochondrial membrane of brown adipose tissue [37]. In addition, functional annotation using Ingenuity Pathway analysis and KnowledgeBase revealed the highest enrichment of DN regulated genes in oxidation-reduction reaction and lipid biosynthesis pathways including the genes Srd5a2, Scd1 (Stearoyl-CoA desaturase), HSD17B11 (Estradiol 17-beta-dehydrogenase 11), FASN (Fatty acid synthase), DEGS2 (Delta(4)-desaturase, sphingolipid 2), ALOX15 (Arachidonate 15-lipoxygenase), and PECR (Peroxisomal trans-2-enoyl-CoA reductase) (supplemental data). Scd1, for example, catalyzes the rate-limiting reaction of monounsaturated fatty acid synthesis [38] and is regulated by leptin, a key regulator of obesity-linked diabetes, through insulin independent mechanisms [39] while FASN deregulation is linked to metabolic diseases and insulin-resistance [40]. While clear associations between these genes and DN are not yet elucidated, their involvement in important biochemical pathways of insulin metabolism, oxidative stress, and obesity, suggest they have important influences on renal injury.

We also identified genes whose expression in diabetic animals was affected by P78 treatment. 12.2% (138/1129) of all RNA transcripts showing abnormal expression in DN were reverted back to partial or complete normal levels by treatment. Because P78 is effective in reducing many of the pathological features of DN [24, 25], we propose that this core set of P78 targets may be responsible for the pathology seen in DN and represent key druggable targets.

Non-homologous end-joining factor 1 (Nhej1), for example, had the highest expression increase in DN (>30 fold compared with control) and the highest decrease (>30 fold compared with diabetic) by both early and late P78 treatments. Nhej1 encodes an essential repair factor in double-stranded DNA breaks [41]. Its elevated expression may be a response to diabetes-induced DNA damage in renal proximal tubule cells [42]. The protective role of P78 in reducing assault on the kidney by diabetes-induced DNA damage, may in turn, result in decreased levels of Nhej1, thus, implicating genotoxicity as a mechanism in renal injury.

Ept1 (Ethanolaminephosphotransferase 1) and Selm (Selenoprotein M), genes related to the selenoprotein gene family, were also in the group regulated by both diabetes and P78. Ept1 is involved in the formation and maintenance of vesicular membranes with relevance to golgi function [43], while Selm codes for a selenocysteine containing protein that maintains redox balance and is linked to obesity and amyloid beta aggregation in the brain [44, 45]. Both genes were upregulated by diabetes with expression reversed by treatment. These along with Txnrd1 (Thioredoxin reductase 1) which plays a role in oxidative stress and hyperglycemic events [46] form a cluster of genes with roles in selenium metabolism and oxidative stress that may be linked to renal damage. Ugt1a and Mamdc4, which were downregulated and Cyp4a14 and Kdm4b, which were upregulated in diabetes by 2-4 fold also had near complete restoration of expression levels to control by P78. Ugt1a genetic variants predict high risk for Type 2 diabetes mortality [47] while Mamdc4 deletions are associated with a group of inflammatory axial diseases [48]. Cyp4a14 KO mice have increased hypertension and develop diabetic nephropathy when treated with streptozotocin (STZ) compared to wild-type treated with STZ [49] and Kdm4b, a lysine demethylase, plays a role in regulating chromatin structure [50]. Given their regulation by both diabetes and P78 treatment, and their association with important metabolic functions, these genes are candidate biomarkers with clinical relevance in DN.

We also noted that expression changes in some genes were unique to early or late stage diabetes suggesting that some pathological events/pathways in the diabetic kidney transcriptome are stage-specific. On the other hand since ~64% of the gene changes in the kidney were similar in the two stages of diabetes, it can be argued that most of the pathology in DN are initiated at a very early stages of the disease. Intervention at either stage with P78 is likely to reduce diabetic insult to the kidney, as we have previously shown [25], through its regulation of these genes. Genes regulated by diabetes and not reversed by treatment are likely to be important as well and may be clustered in yet unknown pathways, which could represent metabolic responses to hyperglycemia that may or may not result in deleterious pathological consequences.

IPA analysis showed mitochondrial dysfunction as the most significantly regulated pathway by diabetes. It was enriched with the highest number (61 genes; 7%) of kidney genes showing expression changes in diabetes. After treatment those numbers were reduced by ~85-90% relative to controls. This analysis strongly implicates mitochondrial dysfunction as a key event in the development of DN, regardless of whether it is a primary or secondary target of diabetes, and provides unequivocal evidence for the utility of P78 treatment in its management. In addition, since treatment reversed several diabetes-induced changes in both early and late stage pathology, the P78 peptide has broader clinical relevance for the disease. Other canonical pathways enriched by diabetes-regulated genes were fatty acid metabolism, Nrf2-mediated oxidative stress, oxidative stress, and those implicated in renal injury, many of which were less populated after treatment.

Non-coding transcripts including miRNA, snRNA, and snoRNA, were some of the most highly regulated molecules in the diabetic kidney. Small RNAs are known to control the transcriptome, kinome and proteome domains and are involved in normal and pathological gene regulation. Many that showed changes in expression with diabetes were also reversed in the direction of these changes after P78 treatment suggesting that members of this group, with functions yet unknown, are important in development and progression of the disease as well as in understanding its future management.

The *Ins2* ^*Akita*^ mouse is a good model of Type I diabetes. However, it will be interesting to see how many of the diabetes-induce gene expression changes we have documented are common to Type II diabetes and how many are found in other diabetic complications such as diabetic retinopathy.

## Conclusion

Diabetes altered expression of a group of coding and non-coding genes in the D2.B6-*Ins2* ^*Akita*^/MatbJ model of DN. Treatment of diabetic mice with P78 reversed expression of a specific subset of these genes. These may be key regulators of diabetes-induced kidney dysfunction as P78 was previously shown to reduce physiological and pathological features of DN. While the precise roles of these genes in DN pathology remain unclear, they give initial clues to how the disease develops. Our study provides a set of unique biomarkers to be exploited in DN and other diabetic complications and suggests that the PEDF-P78 peptide holds promise for the management of this disease.

## Abbreviations

ACE: Angiotensin-converting enzyme
DEG: Differentially expressed genes
DN: Diabetic nephropathy
ESRD: End-stage renal disease
FPKM: Fragments per kilobase of exon per million fragments mapped
IPA: Ingenuity pathway analysis
PEDF: Pigment epithelium-derived factor
VEGF: Vascular endothelial growth factor
